# Methods and Tools Used for Biosecurity Assessment in Livestock Farms in Africa: A Scoping Review

**DOI:** 10.1155/2024/5524022

**Published:** 2024-04-16

**Authors:** Ronald Vougat Ngom, Andrea Laconi, Mohamed M. M. Mouiche, Gaspard J. Ayissi, Adonis M. M. Akoussa, Stephane D. Ziebe, Giuditta Tilli, Henriette A. Zangue, Alessandra Piccirillo

**Affiliations:** ^1^School of Veterinary Medicine and Sciences, University of Ngaoundere, Ngaoundere 454, Cameroon; ^2^Department of Comparative Biomedicine and Food Science, University of Padua, 35020, Legnaro, Italy; ^3^National School of Agro-industrial Sciences Ngaoundere, Ngaoundere 454, Cameroon

## Abstract

Farm biosecurity has gained increasing attention worldwide during the last decades because of its role in reducing the occurrence of diseases and improving animal performance. Recently, recommendations to reinforce the concept of farm biosecurity in lower- and middle-income countries have been advised. Therefore, this review aims to provide a comprehensive description of the methods and tools used to assess biosecurity compliance in livestock farms in Africa and formulate recommendations. The present review followed the Preferred Reporting Items for Systematic Reviews and Meta-Analysis extension for scoping reviews guidelines. Peer-reviewed studies reporting biosecurity assessment in poultry, cattle, pig, goat, or sheep farms in Africa were included. Five databases were searched with no date restrictions. A total of 41 studies across 17 countries were finally selected. Selected studies were all published after 2008, and an increasing trend in the number of papers published per year was noticed. In total, 41 different methods for biosecurity assessment were found to be used in African countries, meaning that even within the same country, the same animal species, and the same farming system, different methods were utilized. In many papers, the methods used for biosecurity evaluation were poorly described. In addition, during the biosecurity assessment, measures related to the purchase of laying hens, egg transport and management, calves, calving and dairy management, and nursery units were almost not considered. These measures should be contemplated in future studies since they are related to important risk factors for the introduction and dissemination of infectious diseases. Interestingly, some measures not considered in European biosecurity tools were identified in the selected studies. The observed high difference in methods used may be due to the lack of regulations on biosecurity in African countries; therefore, the authors recommend the development and implementation of a harmonized and contextualized method for the assessment of biosecurity in livestock farms in Africa.

## 1. Introduction

Worldwide, the livestock sector significantly contributes to the economy of many countries and plays an important role for both food security and employment [1, 2]. The high density of animal production sites and their related contact structures are drivers for infectious animal diseases and are responsible for livestock morbidity and mortality, as well as economic losses [3]. Animal infectious diseases represent one of the main hindrances in the livestock sector in Africa [4]. In many cases, the lack of effective implementation and enforcement of guidelines on how to deal with infectious diseases and pests has led to the collapse of industries at local and regional levels. Indeed, livestock diseases, such as foot-and-mouth disease (FMD), peste des petits ruminants, avian influenza (AI), and African swine fever (ASF) are among the most important with serious economic impacts on farmers and the entire sector. In Ethiopia, in addition to the severe social impact, it has been estimated that the economic loss caused by FMD due to bulls' rejection from the international market could be above 3 million USD [5]. Additionally, the average annual pig production loss due to an ASF outbreak in Nigeria was estimated to range between 649,000 and 94,539,870,064 USD [6]. Even more worryingly, the economic losses due to a potential national epidemic of AI in Nigeria were estimated to be over 2.2 billion USD [7].

The introduction of infectious agents in a farm may have severe implications beyond the farm level, including increasing antimicrobial usage (AMU) and the development of antimicrobial resistance (AMR) that can afterward be transmitted to humans [8]. This can be associated with an increased impact on population health and the economy. Indeed, it is well known that AMU is the main driver of AMR [9], a serious public health concern [10] with a high death rate recorded in Africa [11]. It is, therefore, of great importance for livestock farmers to prevent diseases rather than to cure them. Since prevention based on the prophylactic use of antimicrobials [12] should be avoided.

Biosecurity represents a key strategy to reduce the occurrence of infectious diseases in livestock productions. Indeed, farms' biosecurity has gained increasing attention during the last decades because of its importance in animal production [13]. Due to this, a Progressive Management Pathway approach for terrestrial animal biosecurity was developed by the Food and Agriculture Organization of the United Nations (FAO) to strengthen biosecurity in terrestrial animal productions and value chains [14]. Strengthening biosecurity is one of the key thematic components of the One Health priority program area of the FAO Strategic Framework's aspiration of “Better Production.” FAO defines the term biosecurity as “a strategic and integrated approach to analyzing and managing risks to human, animal, and plant life and health, and associated risks to the environment” [15]. For the World Organization for Animal Health, it is “a set of management and physical measures designed to reduce the risk of introduction, establishment, and spread of animal diseases, infections or infestations to, from and within an animal population” [16]. In the context of animal production systems, a recent review [17] defines a biosecurity measure as “the implementation of a segregation, hygiene, or management procedure (excluding medically effective feed additives and preventive/curative treatment of animals) that specifically aims at reducing the probability of the introduction, establishment, survival, or spread of any potential pathogen to, within, or from a farm, operation or geographical area.” “At the farm level, biosecurity measures may focus either on reducing the risk of entry of new pathogens (external biosecurity) or on reducing the internal dissemination of pathogens (internal biosecurity)” [18]. Appropriate biosecurity practices have been demonstrated to be related to improved performance, quality of animal production, better financial return for farmers, and low AMU [19, 20].

A well-known system (Biocheck.UGent™) has been developed and successfully used in several countries worldwide to assess biosecurity in intensive livestock farms [14, 21–24]. However, the implementation of farm biosecurity measures varies widely according to many factors [25, 26], including geographic region, social groups, farmers' sociodemographics and attitudes, access to information, etc. [27, 28].

In the majority of African countries, there is no specific regulation on biosecurity in livestock farms. Then, the question is, which methods and tools, if any, are used to assess biosecurity in Africa? In line with the recommendations of a recent review [8], in order to reinforce the concept of farm biosecurity in lower- and middle-income countries, this review aims to provide a comprehensive description of the methods and tools used to assess biosecurity in livestock farms in African countries and formulate recommendations according to the outcomes of the results found. The “methods” or “tools” here are referred to the standardized process/system through which biosecurity (or management) was evaluated.

## 2. Materials and Methods

This scoping review was performed as described in the Levac et al. [29] and reported according to the Preferred Reporting Items for Systematic Reviews and Meta-Analysis extension for scoping reviews [30].

### 2.1. Protocol and Registration

A scoping review protocol was first developed and stored in the University of Padua Research Archive institutional repository (https://www.research.unipd.it/handle/11577/3495042). The protocol was also registered online in the Systematic Reviews for Animals and Food (SYREAF) website (https://www.research.unipd.it/retrieve/dd025c7d-acb2-4cad-bae6-03a5d5769048/Scoping%20Review%20Protocol-Biosecurity%20measure%20assessment%20in%20animal%20in%20Africa_final).

### 2.2. Eligibility Criteria

Peer-reviewed studies available in English or French reporting biosecurity assessment in livestock farms in Africa were eligible for inclusion. Due to their importance in the African context in terms of livestock production [31], only studies assessing biosecurity in poultry (limited to broilers, layers, ducks, turkeys, or geese), cattle, pigs, goats, and sheep were included.

### 2.3. Information Sources

CAB Abstracts (Ovid interface), Agricola (in Proquest), Web of Science (WOS), Scopus, and PubMed, available via access of the University of Bern (Switzerland), were chosen as databases and searched with no date restrictions. The search was performed between the 17th and 21st August 2023. All available databases in WOS were used (Web of Science Core Collection, ProQuestTM Dissertation and Theses Citation Index, KCI-Korean Journal Database, Medline, Preprint Citation Index, and SciELO Citation Index) except for Arts and Humanities Citation Index, Conference Proceedings Citation Index-Science, and Conference Proceedings Citation Index-Social Science and Humanities, since their research focus was not within the aim of this scoping review.

### 2.4. Search Strategy

As described by Higgins et al. [29], the search strategy included a multistrand approach that uses a series of searches with different combinations of concepts to gather all possibly related research, achieving thus high sensitivity. The search string formatting was adapted to each database to comply with the specific requirements of each database.

The concept of the search strategy was the following: (Biosecurity) AND (Farm) AND (Cattle or poultry or pigs or goats or sheep) AND (African countries). *Supplementary 1* shows the search strategy applied in all databases.

### 2.5. Selection Process

All citations retrieved in the literature search were imported into Zotero (version 6.0.27) software for deduplication. After duplicate removal, the records obtained were uploaded and screened in Rayyan (https://www.rayyan.ai/). At each stage, six independent reviewers performed the screening to reduce the possibility of excluding relevant records. One-third of the citations were assigned to each pair of reviewers to guarantee that each citation would have been screened by two independent reviewers.

First, the selection process consisted of title and abstract screening. To increase consistency among all reviewers, a calibration exercise was performed by screening 60 randomly selected studies. This calibration exercise enabled discussion and solved disagreements before carrying out the full selection process [32]. Eligibility of the studies was assessed with the following questions: (i) Does the study concern at least one of the following species: poultry, cattle, pigs, goats, and sheep? (ii) Is the study an original research? (iii) Does the study occur in at least one African country? (iv) Does the study concern biosecurity assessment?

Possible answers to the above questions were: “Yes,” “No,” or “Unclear.” For a study to be included, the response to all four questions had to be “Yes” and/or “Unclear.” Any study with “No” as an answer to any of the questions was excluded. Articles selected for full-text screening were obtained using PubMed and WOS via the library of the University of Padua.

After a second calibration exercise (performed by all reviewers) on 30 randomly selected papers among those included in this phase, the following questions were used for inclusion during the full-text screening: (i) Is a full text available? (ii) Is a full text of more than 500 words available? (iii) Is the full text available in English or French? (iv) Does the study concern biosecurity assessment at the farm level? (v) Does the study concern the assessment of biosecurity measures related to a specific disease/pathogen? (vi) Is the study observational in design? (vii) The question “Does the study provide the total level of biosecurity of the farm?” initially included in the protocol was removed after the calibration phase.

Possible answers to the above questions were: “Yes” or “No.” Except for the question (v), for a study to be included, the response to all other questions had to be “Yes.” Any study with “No” (except for question (v)) as an answer for any questions was excluded. For both screening phases, when a consensus between a pair of reviewers was not reached, a third reviewer was asked to solve the conflict.

### 2.6. Data Charting Process

Data charting was conducted in a standardized Excel (version 2013) form developed by the first author and validated by all authors by performing a calibration exercise in which data were extracted from eight randomly selected papers [33]. Similar to the screening phase, each pair of independent reviewers performed data extraction of one-third of the included papers. If necessary, a third author helped to solve the uncertainties among reviewers.

### 2.7. Data Items

From each paper, the following items were extracted: location (i.e., country), year of publication, study design, animal species investigated, methods or tools used to assess biosecurity, measures evaluated, etc. The standardized Excel form with all variables can be found in *Supplementary 2*.

### 2.8. Data Analysis

The results of the literature search were reported, including the number of citations screened, duplicates removed, and full-text documents screened. A flow diagram that details the reasons for exclusion at the full-text level was provided. Characteristics of the included studies were narratively summarized after tabulation. Microsoft Excel 2013 was used to create histograms. The tools or methods used for biosecurity assessment were described. The biosecurity measures evaluated per species were classified according to Biocheck.UGent™ (https://biocheckgent.com/en/) and narratively synthesized. External or internal biosecurity measures were grouped. A map was created using Qgis 3.30 to show the geographical distribution of the included studies.

## 3. Results

### 3.1. Study Selection

**Figure 1:**
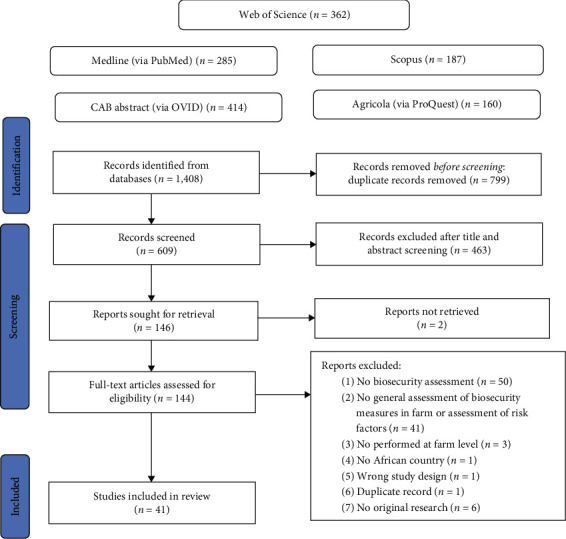
Flow diagram illustrating the selection process of eligible studies. This flow diagram presents the selection process and the number of papers selected in each step.

A total of 1,408 citations were identified in the five databases. After the removal of duplicates, a total of 609 articles were retrieved. By applying inclusion and exclusion criteria during the title and abstract screening phase, 146 were selected. At the end of the selection process, 41 studies were found to be eligible (Figure 1). They were all focused on biosecurity assessment on cattle, poultry, pig, goat, or sheep farms in at least one African country.

### 3.2. Studies Characteristics

**Figure 2:**
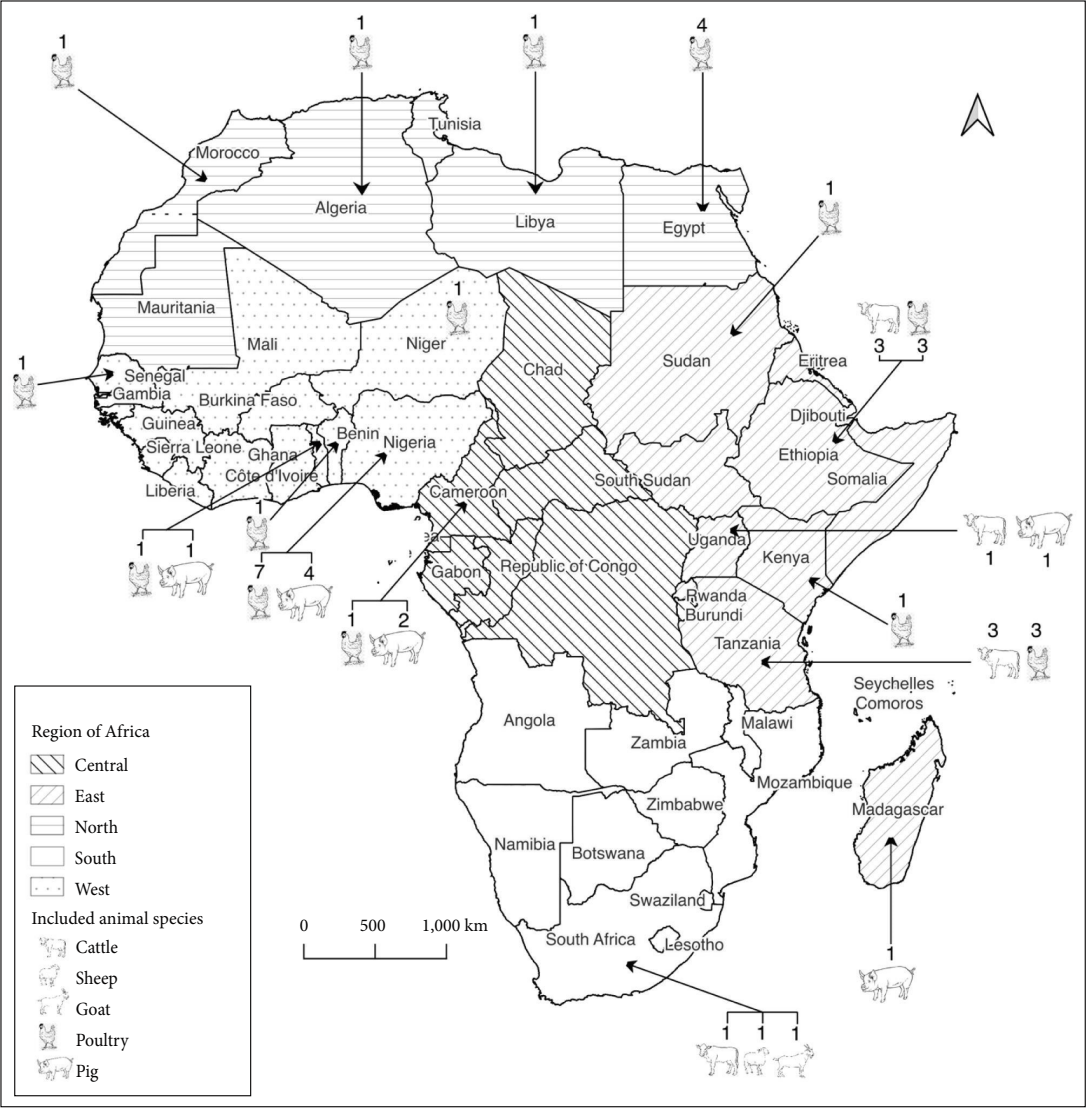
Map of Africa showing for each country, the number of studies per animal species included in the scoping review. The map also presents the number of selected studies per African region.

**Figure 3:**
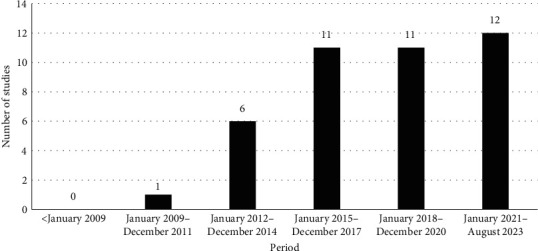
Number of studies on biosecurity assessment in livestock farms published per year in Africa. The figure presents the number of studies published. The search was performed between the 17th and the 21st August 2023 in five databases with no date restriction.

The included studies (*n* = 41), all written in English, were carried out in 17 African countries (Figure 2). Most of the papers referred to countries located in West (*n* = 16) and East (*n* = 14) African regions, with Nigeria (*n* = 11) and Ethiopia (*n* = 6) being the countries with the highest number of studies. All studies were published after 2008, and since then an increasing trend in the number of papers published per year was noticed (Figure 3). Among the 41 studies, one included both pig and poultry farms, while another one considered all three ruminant species. The majority of the studies (*n* = 27) concerned poultry farms. The type of farming system was not reported in 14 papers and 16 studies were performed in mixed systems. Two studies were longitudinal and 39 cross-sectional in design. Random sampling (*n* = 19) was the method most commonly used for farm selection. The number of farms in the included studies varied from 1 to 709, with the majority of studies (*n* = 24) investigating less than 100 farms (Table 1).

### 3.3. Methods and Tools Used for Biosecurity Assessment in Livestock Farms in Africa

Table 2 summarizes the methods and tools used to assess biosecurity implementation in livestock farms in Africa. Out of the 41 studies included in this review, 41 different methods were identified, of which 27 were used for biosecurity assessment in poultry farms, nine in pig farms, six in cattle farms, and only one method in goat and sheep farms. Therefore, different methods of biosecurity assessment were identified not only between countries and between farming systems but also within the same country and within the same farming system. In many papers, the methods used for biosecurity assessment were poorly described. Indeed, most of the studies (*n* = 21) did not report any detail regarding the person(s) performing the evaluation (e.g., age, education, employment, expertise) and/or the respondent(s) (*n* = 10). Considering the studies reporting information about the respondents, it was found that different stakeholders were interviewed; in detail, in 26.8% (*n* = 11) of the studies, the interviewees were only farm owners, in 24.4% (*n* = 10) a combination of three different farm stakeholders, in 17.3% (*n* = 7) either farm owners or farm managers, and in 7.3% (*n* = 3) only farm managers. Biosecurity assessment was mainly carried out through interviews (*n* = 36), followed by focus groups (*n* = 3), and online interviews (*n* = 1). Only one study did not clearly state the type of survey performed. Face-to-face interviews were carried out in 78% (*n* = 32) of the studies, and 53.7% (*n* = 22) of the papers reported that data collected using a survey questionnaire were complemented with direct observations on the farms. The tool used for the survey was not reported in more than 40% (*n* = 17) of the studies; when stated, data were usually collected by using paper support (*n* = 18) and only in a few instances by using digital applications (*n* = 6). The duration of the survey was reported only in one study.

Almost all studies (*n* = 40) reported the level of implementation of individual biosecurity measures in the farms. Descriptive statistical analysis (percentage of farms implementing a specific measure) was mainly used (*n* = 37) for data analysis and presentation of the results. In detail, the implementation of each biosecurity measure was as a binary outcome (“1” if implemented and “0” if not), thus providing the level of implementation of each biosecurity measure in the farms considered. Only in a few studies (*n* = 3), biosecurity assessment was carried out using a scoring system based on weighted biosecurity measures (e.g., low, moderate, and high), and only one study used risk models providing probability estimates for biosecurity evaluation. To conclude, one study sent the results of the assessment to the farm stakeholders to allow for improvement in biosecurity implementation (Table 3).

### 3.4. Biosecurity Measures Evaluated

#### 3.4.1. External Biosecurity

More than 75% of the studies carried out in poultry farms evaluated biosecurity measures related to infrastructure and access to the farms by farmworkers and visitors. Biosecurity measures related to biological vectors, location of the farm, and disposal of manure and carcasses were assessed in 50%–75% of the studies. Biosecurity measures concerning the purchase of day-old chicks, feed and water, material suppliers, and depopulation of the poultry house were assessed in less than 50% of the papers. Meanwhile, only two papers assessed biosecurity measures regarding egg transport, and only one paper assessed the purchase of laying hens.

Biosecurity measures concerning the purchase of animals and reproduction of purchased animals, as well as respect of quarantine or other entry protocols for new animals, were the most investigated (>75%) in ruminant farms. Biosecurity measures concerning pest (and other animals) control, access to the farm by visitors and farmworkers and feed, water diseases transmission were evaluated in 50%–75% of the studies. Measures related to the removal and transport of carcasses were taken into consideration only in two studies.

In the studies concerning pig farms, the external biosecurity measures considered in almost all the papers were related to visitors and farmworkers and the purchase of new animals and semen. Measures concerning the location of the farm and feed, water, and equipment supply were assessed in 50%–75% of the studies. Biosecurity measures regarding pest and birds' control, transport of animals, and the removal of carcasses and manure were evaluated in less than half of the included studies.

Furthermore, several studies investigated external biosecurity measures that are not considered in Biocheck.UGent™, such as stakeholders' level of knowledge and/or training, as well as sources of drinking water.

#### 3.4.2. Internal Biosecurity

In poultry farms, the evaluation of internal biosecurity measures seemed to focus mainly (50%–75% of studies) on disease management and cleaning and disinfection. Meanwhile, biosecurity measures related to materials and measures between compartments were assessed in less than 25% of the studies. Measures concerning egg management were evaluated only in two papers.

Internal biosecurity measures related to work organization and equipment and health management were assessed in more than 75% of the studies carried out in ruminant farms. Measures concerning the management of adult cattle were evaluated in half of the studies. Although more than half (*n* = 4) of the studies on cattle focused on dairy farms, only one evaluated biosecurity measures related to calves, calving, and dairy management (milking techniques and management).

In pig farms, the biosecurity measures most commonly investigated concerned cleaning and disinfection, followed by measures concerning disease management, measures between compartments, and the use of equipment. Biosecurity measures concerning farrowing and suckling periods and finishing units were both evaluated in just one study. None of the included studies assessed biosecurity in the nursery unit.

Additional internal biosecurity measures not considered in Biocheck.UGent™ but assessed in the included studies, mainly concerned veterinary care and treatment protocols.

### 3.5. Other Factors Assessed for Their Association with Biosecurity Measures in Farms

**Figure 4:**
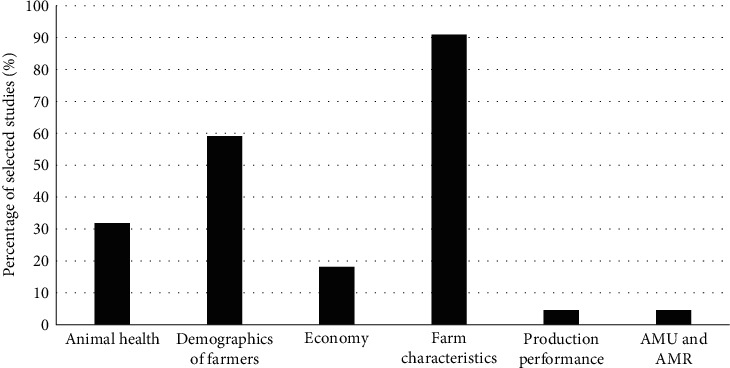
Factors assessed for their association with biosecurity measures in livestock farms in Africa (*n* = 22). The figure reports the number of studies in which the association between at least one biosecurity measure and another factor (i.e., animal health, demographics of farmers, economy, farm characteristics, production performance, antimicrobial usage (AMU), and antimicrobial resistance (AMR)) was assessed. In many studies, more than one factor was considered.

**Figure 5:**
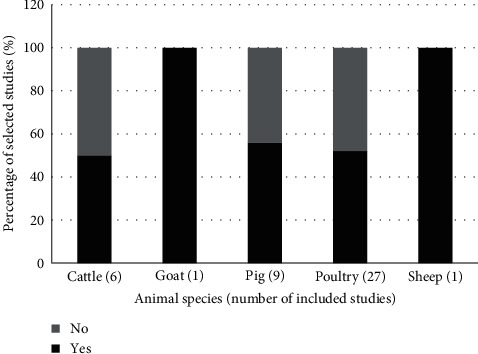
Number of studies assessing the impact of biosecurity measures in Africa. The figure reports the number (percentage) of studies per animal species in which the association between at least one biosecurity measure and another factor (i.e., animal health, demographics of farmers, economy, farm characteristics, production performance, antimicrobial usage, and antimicrobial resistance) was assessed. In many studies, more than one factor was considered. One study considered all the three ruminant species and one both pig and poultry farms.

In 22 of the selected studies (53.7%), the association between biosecurity implementation and other factors (e.g., sociodemographics of farm stakeholders, farm characteristics, production performance, animal health status, AMU, and resistance) was evaluated. The majority of the papers investigated the relationship between biosecurity implementation and farm characteristics (*n* = 20) and/or stakeholder's sociodemographics (*n* = 13) (Figure 4). In 55.6% and 51.9% of the studies carried out in pig (*n* = 9) and poultry (*n* = 27) farms, respectively, the association between biosecurity practices and those factors was evaluated (Figure 5). In 91% (*n* = 20) of these studies, the association of the abovementioned factors with biosecurity measures was assessed through descriptive analysis. The other two studies used simulation models.

## 4. Discussion

This review aimed to provide a comprehensive description of the methods and tools used to assess biosecurity in livestock farms in Africa and formulate recommendations. The results of this scoping review showed that only 41 studies have been carried out throughout the whole African continent to assess biosecurity compliance in poultry, cattle, pig, goat, or sheep farms. All included studies were performed in 17 African countries. This means that no published studies were available for 68.5% (*n* = 37) of African countries. In contrast with other parts of the world, where many studies on biosecurity have been undertaken since the last century [75, 76], the studies included in this review were published after 2008; however, since then, we noticed an increasing trend in the number of publications on farm biosecurity assessment in African countries. This finding seems to indicate an increasing interest and awareness on the impact of biosecurity in reducing the emergence and spread of animal infectious diseases, including zoonoses, which can cause high morbidity and mortality in both humans and animals and result in substantial economic losses [77–79], especially in countries where the number of people facing hunger is constantly increasing [80]. Indeed, the improvement of livestock production and productivity is particularly important in countries where the demand for food of animal origin is increasing due to the rapid growth of the population [81]. The increasing number of biosecurity studies may also be linked to the rapid modernization of the livestock sector in Africa. Indeed, improving livestock productivity has the potential to substantially improve food security and alleviate poverty in Africa. In the context of the African continent, where there is a lack of strict regulations and policies on livestock production (e.g., disease surveillance, feed distribution, veterinary drug market, etc.), biosecurity can be very helpful in reducing the impact of animal diseases and therefore the amount of antimicrobial drugs used in the veterinary sector.

Most of the studies selected concerned poultry farming (65.9%). This result is consistent with the findings of other reviews on livestock production in Africa [33, 82], highlighting the importance of the poultry sector in this continent [1, 83]. Indeed, the poultry sector led the livestock production in Africa, accounting for roughly 2.1 billion animals in 2020 [84]. In addition, poultry consumption led by Asian countries in the last decade is projected to grow rapidly in sub-Saharan Africa [85], reflecting the importance of poultry production in this part of the world.

The findings of this scoping review showed that 41 different methods were used to assess biosecurity compliance in livestock farming in African countries. Within the same country, the same animal species, and the same farming system, different methods were used. The high diversity of methods may be due to the lack of regulations on farm biosecurity, even if the majority of African countries have a general legislation on livestock production [86, 87].

This review identified the need for specific legislation on biosecurity in livestock production in African countries, since biosecurity at the farm level provides the basis for biosecurity in the entire production chain [88]. Furthermore, these legislations would allow to develop an effective and reliable system to assess biosecurity compliance and thus to identify which biosecurity measures should be implemented and/or improved for disease prevention and control. In addition, this system may help to monitor farm biosecurity compliance over time for benchmarking [89] and to estimate the benefits in production and health status generated by the implementation of a given measure [24, 90].

The majority of the studies carried out in Africa used a descriptive evaluation (percentage of farms implementing a specific measure) for the assessment of the implementation of biosecurity measures. This method represents the most simplified way of assessing biosecurity (qualitative assessment). However, since there is no ranking of methods for biosecurity assessment according to their robustness, the method based on descriptive evaluation should still be considered valid. Biosecurity assessment methods based on quantitative methods and tools are also available worldwide. Among these, there are methods that quantify both external and internal biosecurity by considering their relative importance. Biosecurity assessment tools, such as PA-DRAP [91], Biocheck.UGent™ [90], and BioAsseT [92], have been developed in the United States, Europe, and Japan, respectively. Other methods used probability-based risk models for biosecurity assessment [93–95]. Although each method/tool has its own strengths and weaknesses, the ideal one should be tailored to the region where it is applied, considering geographical conditions, farming systems, livestock production (i.e., single farm system vs. integrated system), and sociocultural, demographic, and environmental factors. Hence, the need to develop a harmonized method to assess biosecurity compliance in livestock farming in Africa.

In poultry farms, measures related to the purchase of laying hens, eggs transport, and management were not considered in the majority of the studies. The assessment of these measures should be included in future surveys as they are important risk factors in layer farms. Indeed, the storage temperature of the eggs in the farm or during transportation may influence the proliferation of *Salmonella* on the eggshell and its penetration into the egg content [96]. Observance of good practices during egg handling, including sorting, candling, grading, and packaging, is also important to reduce the risk of contamination [96–98]. Wakawa et al. [99] showed that the exchange of egg crates between traders and farmers and the introduction of traders' egg crates into poultry pens are high-risk factors for highly pathogenic AI.

In the studies performed in ruminants, biosecurity measures related to calves, calving management, and dairy management were rarely evaluated. As the majority of the studies were performed in dairy farms, there is an important gap in biosecurity assessment because calf management has an important effect on animal performance and health [100]. Indeed, diarrhea, one of the main concerns in cattle farming worldwide, resulting in huge economic losses to dairy producers [101–103], can be caused by poor management [104]. Furthermore, the housing of calves [105], cow cleanliness and barns' cleaning [106], time of first colostrum feeding, and colostrum quantity and quality [107, 108] can affect animal health. However, only a few African studies have evaluated these biosecurity practices and these gaps must be covered in future surveys and included in future legislations on livestock production. Indeed, due to their impact, management and environmental factors concerning cattle rearing are regulated by law in Europe [109].

In pig farms, all selected studies focused on measures related to disease management and cleaning and disinfection, but the assessment of biosecurity measures related to farrowing and suckling period and finishing was neglected. Of notice, no study assessed biosecurity in the nursery, although this can be explained by the fact that in Africa the majority of pig farms are farrow-to-finish farms. The lack of information about these important steps of pig production represents another gap that needs to be filled. For example, during gestation, housing (e.g., floor space per sow, pen design, flooring, sow in group or not, etc.) is an important factor that can affect the productivity and health of pigs [110, 111]. In the European Union, the housing system of sows (from week four after confirmation of gestation) is regulated by law (Directive 2008/120/EC) (https://eur-lex.europa.eu/legal-content/EN/ALL/?uri=CELEX%3A32008L0120). Farrowing management can also affect animal health, performance, and welfare [112, 113]. Several studies [114, 115] have shown the effect of the level of attention and care provided to piglets during the first days on their performance and health. Nguyen et al. [116] showed that the presence of staff during farrowing significantly reduced piglet mortality in the neonatal period. Manual positioning of piglets near the udder shortly after birth was associated with reduced mortality [117].

In the selected papers, other measures not included in the Biocheck.UGent™ list were considered during the biosecurity assessment. These may be related to the African context and to specific features of the African livestock farming. For example, many farmers buy and administer veterinary drugs by themselves, due to the low number of veterinarians and the weak regulation on veterinary drugs [118–121]; therefore, in this context, assessing the availability of treatment protocols in the farms and/or the presence of veterinarians during drug administration should be considered. Indeed, the misuse of antimicrobial drugs has been previously reported in studies performed in Africa [118, 119], representing a risk for disease control and underlining the need for the development of biosecurity assessment methods adapted to the African context.

This review showed that half of the papers studied the association between biosecurity compliance and other factors, such as the socio-demographics of farm stakeholders. Indeed, farmers' sociodemographics have been shown to influence biosecurity implementation [27, 28]. Surprisingly, only one study investigated the relationship between biosecurity and livestock production performance. This gap should be addressed as biosecurity not only aims at disease prevention and control but also at improving animal performance. Although the assessment of the direct impact of biosecurity on the technical performance of animals can be difficult, further studies should assess the effectiveness of implementing biosecurity measures by using robust research methodologies.

This scoping review focused on original publications and had limited scope for the inclusion of governmental and non-governmental reports. Even though this can represent a limitation of this study, during the full-text screening of the 144 selected studies, none of them reported the presence of any biosecurity regulation/legislation in Africa. This finding seems to support the fact that this review may be representative of the actual state of biosecurity assessment at the farm level in Africa.

### 4.1. Recommendations

Based on the findings of this scoping review, the authors recommend to each African country to:

- develop biosecurity legislations that dictate the mandatory implementation of specific biosecurity measures at the farm level to prevent and control diseases and to improve animal production;
- develop a contextualized and harmonized methodology to assess biosecurity in livestock farming;
- raise awareness and train both advisors and farmers on biosecurity.

## 5. Conclusion

The findings of this scoping review show the poor level of harmonization of methods and tools used for biosecurity assessment in livestock farming in Africa and highlight the need for adopting a specific regulation/legislation on biosecurity in livestock production in Africa. Biosecurity compliance in farms provides the foundation for biosecurity in the entire food production chain. This legislation would allow to develop an effective system for on-farm biosecurity assessment. Such a system would be used to prioritize which biosecurity measures should be implemented or improved for disease prevention and control. These measures must be practical, affordable, cheap, and acceptable to producers in the African context.

## Figures and Tables

**Table 1:** General characteristics of the included studies.

Variables	Number of studies	Percentage (%)	References
Language	41	—	—
*English*	41	100	[34–74]
*French*	0	0	—
Region of Africa	41	—	—
*Center*	3	7.3	[52, 54, 62]
*East*	14	34.1	[37–41, 46, 51, 53, 55, 56, 63, 70, 71, 74]
*North*	7	17.1	[36, 44, 48, 58, 64, 69, 72]
*South*	1	2.4	[65]
*West*	16	39.0	[34, 35, 42, 43, 46, 47, 49, 50, 57, 59–61, 66–68, 73]
Study design	41	—	—
*Cross-sectional*	39	95.1	[34–54, 56–59, 61–74]
*Longitudinal*	2	4.9	[55, 60]
Sampling strategy for farm selection	41	—	—
*Multistate*	6	14.6	[34, 41, 49, 50, 57, 72]
*Random*	11	26.8	[42, 44, 45, 47, 52, 56, 59–61, 65, 66]
*Random systematic*	3	7.3	[50, 53, 70]
*Snow-ball*	8	19.5	[36, 54, 62, 67, 68, 71, 73, 74]
*Not reported*	13	31.7	[35, 37–40, 43, 46, 48, 55, 58, 63, 64, 69]
Number of farms included in the study	41	—	—
*≤50*	12	29.3	[35, 37, 38, 40, 46, 47, 53, 55, 60, 63, 64, 69]
*51–100*	12	29.3	[36, 39, 41, 43, 50, 54, 57, 58, 61, 62, 67, 71]
*101–150*	5	12.2	[42, 48, 49, 51, 52]
*151–500*	8	19.5	[44, 56, 59, 65, 68, 72–74]
*>500*	4	9.7	[34, 45, 66, 70]
Animal species	41	—	—
*Cattle*	5	12.2	[40, 51, 55, 56, 65]
*Pig*	8	19.5	[34, 41, 42, 54, 60–62, 67]
*Poultry*	26	63.4	[35–39, 43–50, 52, 53, 57–59, 63, 64, 66, 69–73]
*Poultry and pig*	1	2.4	[68]
*Cattle*, *goat*, *and sheep*	1	2.4	[65]
Poultry species	27	—	—
*Broilers*	5	18.5	[36, 38, 44, 52, 71]
*Ducks*	1	3.7	[69]
*Layers*	1	3.7	[64]
*Broilers and layers*	6	22.2	[37, 43, 50, 57, 63, 73]
*More than two species*	4	14.8	[35, 53, 58, 59]
*Not reported*	10	37.0	[39, 45–49, 66, 68, 70, 72]
Cattle	6	—	—
*Dairy*	3	50.0	[55, 56, 74]
*Beef*	1	16.7	[40]
*Dairy and beef*	1	16.7	[51]
*Not reported*	1	16.7	[65]

**Table 2:** Methods and tools used to assess biosecurity in livestock farms in Africa.

Variable	Pigs		Cattle		Poultry		Poultry and pigs		Cattle, goats, and sheep		Total	
	*N*	(%)	*N*	(%)	*N*	(%)	*N*	(%)	*N*	(%)	*N*	(%)
Interviewer												
Researchers	2	25.0	2	40.0	5	19.2	1	100	0	0	10	24.4
Veterinary officers	0	0	0	0	3	11.5	0	0	0	0	3	7.3
Trained person	1	12.5	1	20.0	2	7.7	0	0	0	0	4	9.8
Others (combination of above)	0	0	1	20.0	2	7.7	0	0	0	0	3	7.3
Not reported	5	62.5	1	20.0	14	53.8	0	0	1	100	21	51.2
Respondent												
Farm owners	2	25.0	1	20.0	8	30.8	0	0	0	0	11	26.8
Farm managers	0	0	0	0	3	11.5	0	0	0	0	3	7.3
Farm owner and farm manager	2	25.0	2	40.0	2	7.7	0	0	1	100	7	17.1
Combination of more than 2 farm stakeholders	1	12.5	2	40.0	7	26.9	0	0	0	0	10	24.4
Not reported	3	37.5	0	0	6	23.1	1	100	0	0	10	24.4
Type of survey												
Interview	6	75.0	5	100	23	88.6	1	100	1	100	36	87.9
Online	0	0	0	0	1	3.8	0	0	0	0	1	2.4
Interview and focus group	2	25.0	0	0	1	3.8	0	0	0	0	3	7.3
Not reported	0	0	0	0	1	3.8	0	0	0	0	1	2.4
Face-to-face survey												
Yes	7	87.5	2	40.0	22	84.7	1	100	0	0	32	78.0
No	1	12.5	2	40.0	1	3.8	0	0	1	100	5	12.2
Not reported	0	0	1	20.0	3	11.5	0	0	0	0	4	9.8
Implementation with direct observation												
Yes	5	62.5	2	40.0	14	53.8	1	100	0	0	22	53.7
No	1	12.5	1	20.0	1	3.8	0	0	1	100	4	9.8
Not reported	2	25.0	2	40.0	11	42.3	0	0	0	0	15	36.6
Support used for the survey												
Digital application	0	0	1	20.0	3	11.6	1	100	1	100	6	14.6
Paper	6	75.0	3	60.0	9	34.6	0	0	0	0	18	43.9
Not reported	2	25.0	1	20.0	14	53.8	0	0	0	0	17	41.5
Duration of the survey (hr)												
1	0	0	0	0	1	3.8	0	0	0	0	1	2.4
Not reported	8	100	5	100	25	96.2	1	100	1	100	40	97.6

Interview was usually performed with a questionnaire with or without additional discussion.

**Table 3:** Data collected during biosecurity assessment in livestock farms in Africa.

Variable	Pigs		Cattle		Poultry		Poultry and pigs		Cattle, goats, and sheep		Total	
	*N*	(%)	*N*	(%)	*N*	(%)	*N*	(%)	*N*	(%)	*N*	(%)
Assessment concerned												
Aggregated biosecurity measures	0	0	0	0	1	3.8	0	0	0	0	1	2.4
Individual biosecurity measure	8	100.0	5	100.0	25	96.2	1	100.0	1	100.0	40	97.6
Methods used												
Scoring method based on weighting biosecurity measures	0	0	0	0	3	11.5	0	0	0	0	3	7.3
Descriptive evaluation (*percentage of farms*)	8	100.0	4	80.0	23	88.5	1	100.0	1	100.0	37	90.3
Probability estimates based on risk models	0	0	1	20.0	0	0	0	0	0	0	1	2.4
Feedback of the results sent to the respondent as a recommendation												
Yes	0	0	0	0	1	3.8	0	0	0	0	1	2.4
No	8	100.0	5	100.0	25	96.2	1	100.0	1	100.0	40	97.6

## Data Availability

The protocol of this review can be found in the University of Padua Research Archive institutional repository (https://www.research.unipd.it/handle/11577/3495042) or in the Systematic Reviews for Animals and Food website (https://www.syreaf.org/protocol/). The standardized Excel form with all variables extracted is provided as supplement.

## Supplementary Materials

Supplementary 1 Search strategies performed in each database.

Supplementary 2 Standardized Excel form used for data extraction.
