# Supplementary material for: Methods and Tools Used for Biosecurity Assessment in Livestock Farms in Africa: A Scoping Review
Source: Transbound Emerg Dis. 2024 Apr 16;2024:5524022. doi: 10.1155/2024/5524022 (PMC12016981; doi:10.1155/2024/5524022)
Supplement: Supplementary 1 — Search strategies performed in each database. [file 5524022.f1.pdf]

Study ID	Author	Year of publication	Journal name

Language of publication	Country where the study took place

Study design (cross sectional, longitudinal)	Animal species study Level1 (Cattle, poultry, Pig, Goa

Animal types studies Level 2 (heifer, fattening Pig, Broiler, backyard poultr	Sample size (nber of farms)

Sample strategy for farm selection (ran

Animal production system (Intensive, semi-intensive, extensive, Smallholders, Mixed, NR)

The assessment concern aggre	Who administ	Field study of	Who respond	Type of surve	Survey face to

Survey with o	if no with visi	if face to face	Survey done u	Survey done u	Use of estima	Use of Scoring

Use of descrip	Is there feedb	Impact of bios	If yes, impact	How the impa	Any comments
