# Supplementary material for: Methods and Tools Used for Biosecurity Assessment in Livestock Farms in Africa: A Scoping Review
Source: Transbound Emerg Dis. 2024 Apr 16;2024:5524022. doi: 10.1155/2024/5524022 (PMC12016981; doi:10.1155/2024/5524022)
Supplement: Supplementary 2 — Standardized Excel form used for data extraction. [file 5524022.f2.docx]

**Search strings performed in each database**

**CAB Abstracts**

1 (biosecurity or 'farm biosecurity' or 'animal biosecurity' or 'preventive veterinary medicine' or 'herd health management').ti,ab.

2 (Farmer or farm* or 'farm-level' or 'farm-level*').ti,ab.

3 (pig* or swine* or pig* or weaner or fattener or sow or piglet* or boar or boars or 'Sus domesticus' or chick* or poultry* or broiler* or layer* or turkey* or duck* or geese or goose or fowl* or avian* or bird* or hen or hens or 'gallus gallus' or flock* or cattle or beef or cow* or calf or calves or 'Bos indicus' or heifer* or bull* or bovine or dairy or zebu or sheep* or caprine or goat* or ovine or ewe or 'small ruminant' or 'food-producing animal*' or 'food animal*' or 'animal husbandry' or 'animal farming' or 'domestic animal*' or livestock).ti,ab.

4 (African or Africa or 'North Africa' or Egypt or Libya or Tunisia or Algeria or Morocco or 'West Africa' or Benin or 'Burkina Faso' or Cameroon or 'Cape Verde' or Chad or 'Ivory cost' or Gambia or Ghana or Guinea or 'Guinea-Bissau' or Liberia or Mali or Mauritania or Niger or Nigeria or Senegal or 'Sierra Leone' or Togo or 'Central African Republic' or Congo or 'Democratic Republic of Congo' or 'Equatorial Guinea' or Gabon or 'Sao Tome' or 'East Africa' or Eritrea or Ethiopia or Somalia or Djibouti or Sudan or 'South Sudan' or Uganda or Kenya or Tanzania or Rwanda or Burundi or Comoros or Mauritius or Seychelles or Madagascar or 'South Africa' or Angola or Botswana or Lesotho or Malawi or Mozambique or Namibia or 'South Africa' or Swaziland or Zambia or Zimbabwe).ti,ab.

5 1 and 2 and 3 and 4

**AGRICOLA**

1 (biosecurity or 'farm biosecurity' or 'animal biosecurity' or 'preventive veterinary medicine' or 'herd health management').ti,ab.

2 (Farmer or farm* or 'farm-level' or 'farm-level*').ti,ab.

3 (pig* or swine* or pig* or weaner or fattener or sow or piglet* or boar or boars or 'Sus domesticus' or chick* or poultry* or broiler* or layer* or turkey* or duck* or geese or goose or fowl* or avian* or bird* or hen or hens or 'gallus gallus' or flock* or cattle or beef or cow* or calf or calves or 'Bos indicus' or heifer* or bull* or bovine or dairy or zebu or sheep* or caprine or goat* or ovine or ewe or 'small ruminant' or 'food-producing animal*' or 'food animal*' or 'animal husbandry' or 'animal farming' or 'domestic animal*' or livestock).ti,ab.

4 (Africa or African or Comoros or Djibouti or Madagascar or Malawi or Seychelles or Cameroon or 'Central African Republic' or Chad or Congo or 'Equatorial Guinea' or 'Atlantic Islands' or Gabon or Morocco or Sudan or Botswana or Lesotho or Swaziland or Benin or 'Burkina Faso' or 'Cape Verde' or Ghana or Guinea or Mauritania or Niger or Senegal or 'Sierra Leone' or Togo or Burundi or Eritrea or Ethiopia or Kenya or Mozambique or Rwanda or Somalia or Tanzania or Uganda or Zambia or Zimbabwe or Angola or Algeria or Egypt or Tunisia or Namibia or 'South Africa' or Gambia or Liberia or Mali or Nigeria or 'Ivory Cost').ti,ab.

5 1 and 2 and 3 and 4

**WEB OF SCIENCES**

#1 TS=(biosecurity OR “farm biosecurity” OR “animal biosecurity” OR “preventive veterinary medicine” OR “herd health management”)

#2 TS=(Farmer OR farm* OR 'farm-level' OR 'farm-level*')

#3 TS=(pig* OR swine* OR weaner OR fattener OR sow OR piglet* OR boar OR boars OR 'Sus domesticus' OR chick* OR poultry* OR broiler* OR layer* OR turkey* OR duck* OR geese OR goose OR fowl* OR avian* OR bird* OR hen OR hens OR 'gallus gallus' OR flock* OR cattle OR beef OR cow* OR calf OR calves OR 'Bos indicus' OR heifer* OR bull* OR bovine OR dairy OR zebu OR sheep* OR caprine OR goat* OR ovine OR ewe OR 'small ruminant' OR 'food-producing animal*' OR 'food animal*' OR 'animal husbandry' OR 'animal farming' OR 'domestic animal*' OR livestock)

#4 TS=(Africa OR African OR Comoros OR Djibouti OR Madagascar OR Malawi OR Seychelles OR Cameroon OR 'Central African Republic' OR Chad OR Congo OR 'Equatorial Guinea' OR 'Atlantic Islands' OR Gabon OR Morocco OR Sudan OR Botswana OR Lesotho OR Swaziland OR Benin OR 'Burkina Faso' OR 'Cape Verde' OR Ghana OR Guinea OR Mauritania OR Niger OR Senegal OR 'Sierra Leone' OR Togo OR Burundi OR Eritrea OR Ethiopia OR Kenya OR Mozambique OR Rwanda OR Somalia OR Tanzania OR Uganda OR Zambia OR Zimbabwe OR Angola OR Algeria OR Egypt OR Tunisia OR Namibia OR 'South Africa' OR Gambia OR Liberia OR Mali OR Nigeria OR 'Ivory Cost')

#1 AND #2 AND #3 AND #4

**PUBMED**

#1 "biosecurity"[Title/Abstract] OR "farm biosecurity"[Title/Abstract] OR "animal biosecurity"[Title/Abstract] OR "preventive veterinary medicine"[Title/Abstract] OR "herd health management"[Title/Abstract]

#2 "Farmer"[Title/Abstract] OR "farm*"[Title/Abstract] OR "farm level"[Title/Abstract] OR "farm level"[Title/Abstract].

#3 "pigs"[Title/Abstract] OR "swine*"[Title/Abstract] OR "pig"[Title/Abstract] OR "weaner"[Title/Abstract] OR "fattener"[Title/Abstract] OR "sow"[Title/Abstract] OR "piglet*"[Title/Abstract] OR "boar"[Title/Abstract] OR "boars"[Title/Abstract] OR "sus domesticus"[Title/Abstract] OR "chick*"[Title/Abstract] OR "poultry*"[Title/Abstract] OR "broiler*"[Title/Abstract] OR "layer*"[Title/Abstract] OR "turkey*"[Title/Abstract] OR "duck*"[Title/Abstract] OR "geese"[Title/Abstract] OR "goose"[Title/Abstract] OR "fowl*"[Title/Abstract] OR "avian*"[Title/Abstract] OR "bird*"[Title/Abstract] OR "hen"[Title/Abstract] OR "hens"[Title/Abstract] OR "gallus gallus"[Title/Abstract] OR "flock*"[Title/Abstract] OR "cattle"[Title/Abstract] OR "beef"[Title/Abstract] OR "cow"[Title/Abstract] OR "cows"[Title/Abstract] OR "calf"[Title/Abstract] OR "calves"[Title/Abstract] OR "bos indicus"[Title/Abstract] OR "heifer*"[Title/Abstract] OR "bull*"[Title/Abstract] OR "bovine"[Title/Abstract] OR "dairy"[Title/Abstract] OR "zebu"[Title/Abstract] OR "sheep*"[Title/Abstract] OR "caprine"[Title/Abstract] OR "goat*"[Title/Abstract] OR "ovine"[Title/Abstract] OR "ewe"[Title/Abstract] OR "small ruminant"[Title/Abstract] OR "food producing animal"[Title/Abstract] OR "food animal"[Title/Abstract] OR "animal husbandry"[Title/Abstract] OR "animal farming"[Title/Abstract] OR "domestic animal"[Title/Abstract] OR "livestock"[Title/Abstract].

#4 "Africa"[Title/Abstract] OR "African"[Title/Abstract] OR "Comoros"[Title/Abstract] OR "Djibouti"[Title/Abstract] OR "Madagascar"[Title/Abstract] OR "Malawi"[Title/Abstract] OR "Seychelles"[Title/Abstract] OR "Cameroon"[Title/Abstract] OR "central african republic"[Title/Abstract] OR "Chad"[Title/Abstract] OR "Congo"[Title/Abstract] OR "equatorial guinea"[Title/Abstract] OR "atlantic islands"[Title/Abstract] OR "Gabon"[Title/Abstract] OR "Morocco"[Title/Abstract] OR "Sudan"[Title/Abstract] OR "Botswana"[Title/Abstract] OR "Lesotho"[Title/Abstract] OR "Swaziland"[Title/Abstract] OR "Benin"[Title/Abstract] OR "burkina faso"[Title/Abstract] OR "cape verde"[Title/Abstract] OR "Ghana"[Title/Abstract] OR "Guinea"[Title/Abstract] OR "Mauritania"[Title/Abstract] OR "Niger"[Title/Abstract] OR "Senegal"[Title/Abstract] OR "sierra leone"[Title/Abstract] OR "Togo"[Title/Abstract] OR "Burundi"[Title/Abstract] OR "Eritrea"[Title/Abstract] OR "Ethiopia"[Title/Abstract] OR "Kenya"[Title/Abstract] OR "Mozambique"[Title/Abstract] OR "Rwanda"[Title/Abstract] OR "Somalia"[Title/Abstract] OR "Tanzania"[Title/Abstract] OR "Uganda"[Title/Abstract] OR "Zambia"[Title/Abstract] OR "Zimbabwe"[Title/Abstract] OR "Angola"[Title/Abstract] OR "Algeria"[Title/Abstract] OR "Egypt"[Title/Abstract] OR "Tunisia"[Title/Abstract] OR "Namibia"[Title/Abstract] OR "south africa"[Title/Abstract] OR "Gambia"[Title/Abstract] OR "Liberia"[Title/Abstract] OR "Mali"[Title/Abstract] OR "Nigeria"[Title/Abstract] OR "ivory cost"[Title/Abstract].

#1 AND #2 AND #3 AND #

**SCOPUS**

( TITLE-ABS-KEY ( biosecurity  OR  "farm biosecurity"  OR  "animal biosecurity"  OR  "preventive veterinary medicine"  OR  "herd health management" )  AND  TITLE-ABS-KEY ( farmer  OR  farm*  OR  "farm-level"  OR  "farm-level*" )  AND  TITLE-ABS-KEY ( pig*  OR  swine*  OR  sow  OR  piglet*  OR  boar  OR  boars  OR  "Sus domesticus"  OR  chick*  OR  poultry*  OR  broiler*  OR  layer*  OR  turkey*  OR  duck*  OR  geese  OR  goose  OR  fowl*  OR  avian*  OR  bird*  OR  hen  OR  hens  OR  'gallus  AND gallus'  OR  flock*  OR  cattle  OR  beef  OR  cow*  OR  calf  OR  calves  OR  "Bos indicus"  OR  heifer*  OR  bull*  OR  bovine  OR  dairy  OR  zebu  OR  sheep*  OR  caprine  OR  goat*  OR  ovine  OR  ewe  OR  "small ruminant"  OR  "food-producing animal*"  OR  "food animal*"  OR  "animal husbandry"  OR  "animal farming"  OR  "domestic animal*"  OR  livestock )  AND  TITLE-ABS-KEY ( african  OR  africa  OR  "North Africa"  OR  egypt  OR  libya  OR  tunisia  OR  algeria  OR  morocco  OR  "West Africa"  OR  benin  OR  "Burkina Faso"  OR  cameroon  OR  "Cape Verde"  OR  chad  OR  "Ivory cost"  OR  gambia  OR  ghana  OR  guinea  OR  "Guinea-Bissau"  OR  liberia  OR  mali  OR  mauritania  OR  niger  OR  nigeria  OR  senegal  OR  "Sierra Leone"  OR  togo  OR  "Central African Republic"  OR  congo  OR  "Democratic Republic of Congo"  OR  "Equatorial Guinea"  OR  gabon  OR  "Sao Tome"  OR  "East Africa"  OR  eritrea  OR  ethiopia  OR  somalia  OR  djibouti  OR  sudan  OR  "South Sudan"  OR  uganda  OR  kenya  OR  tanzania  OR  rwanda  OR  burundi  OR  comoros  OR  mauritius  OR  seychelles  OR  madagascar  OR  "South Africa"  OR  angola  OR  botswana  OR  lesotho  OR  malawi  OR  mozambique  OR  namibia  OR  "South Africa"  OR  swaziland  OR  zambia  OR  zimbabwe ) )
